# Behavioral disorders and cognitive impairment associated with cerebellar lesions

**DOI:** 10.1186/s40303-015-0009-1

**Published:** 2015-05-15

**Authors:** Stefan Grossauer, Katharina Koeck, Thomas Kau, Joerg Weber, Giles H Vince

**Affiliations:** Department of Neurosurgery, Academic Hospital Klagenfurt, Feschnigstrasse 11, Klagenfurt, A-9020 Austria; Department of Diagnostic and Interventional Radiology, Academic Hospital Klagenfurt, Feschnigstrasse 11, Klagenfurt, A-9020 Austria; Department of Neurology, Academic Hospital Klagenfurt, Feschnigstrasse 11, Klagenfurt, A-9020 Austria

**Keywords:** Cerebellar lesions, Neurocognitive deficits, Neuroimaging, Posterior fossa surgery, Neurooncology

## Abstract

In the last decade evidence has accumulated that suggests that the cerebellum is involved not only in motor but also in behavioral and cognitive functions. A myriad of anatomical, clinical and imaging studies support that assumption. The lengthened survival of patients with cerebellar tumors has also brought an increased awareness of neurocognitive deficits to the neurooncological community. Although evidence from neurosurgical case series exists that clearly demonstrates that patients afflicted from posterior fossa tumors are at high risk for long-term cognitive or adaptive deficits, there is still a lack of systematic translational review on this issue. Accordingly a systematic review was conducted to summarize the impact of cerebellar lesions on behavior and cognition. The findings and clinical implications are discussed in the light of the recent advances in neuroimaging techniques.

## Introduction

In the last decade evidence has accumulated that suggests the cerebellum is involved not only in motor but also in cognitive functions [1]. This view is supported by the fact that the cerebellum contains more than half of all the neurons in the brain. Anatomical, clinical and imaging findings demonstrate that the cerebellum is engaged in cognitive and affective functions as well as motor control [2]. While so called eloquent regions of the brain are well defined for the supratentorial brain and the brainstem, the cerebellum remains a more or less low eloquent neural tissue from the neurosurgical perspective. Evidence from converging modalities also indicates that there is a functional topography in the human cerebellum for overt control of movement versus higher cognitive functions. New data support the recently attributed role of the cerebellum as a modulator of the superior mental and social functions [3]. Functional magnetic resonance imaging (fMRI) studies demonstrate that regions active during overt movement differ from those involved in higher-level language, spatial processing and working memory tasks, i.e. overt movement activates sensorimotor cortices along with contralateral cerebellar lobules IV-V and VIII, whereas more cognitively demanding tasks engages prefrontal and parietal cortices along with cerebellar lobules VI and VII. These findings provide further support for a cerebellar role in both motor and cognitive tasks, and better establish the existence of functional subregions in the cerebellum [1].

Positron emission tomography (PET) and fMRI studies show that right cerebellar activation has been reported in verbal fluency paradigms and the site of activation is contralateral to the activation of the frontal cortex. Greater cognitive demands in verbal fluency tasks seem to lead to more extensive cerebellar activation [4].

Evidence from operative series demonstrates that right cerebellar lesions lead to verbal deficits because of the crossed pathways, whereas in left cerebellar lesions spatial deficits occur [4]. Lesions of the vermis were found to be frequently associated with behavioral alterations and executive dysfunction similar to those produced by a disruption of frontal-subcortical circuits, as well as congenital cerebellar abnormalities of childhood that lead to a cascade of abnormal cortical development [5].

Further evidence for functions of the cerebellum has come from examination of cerebellar abnormalities in psychopathological disorders such as schizophrenia, autism, or attention deficit hyperactivity disorder [2,6,7].

The comparatively long survival of patients with tumors of the posterior fossa has brought an increased awareness of neurocognitive deficits to the neurooncological community. In the past, these deficits were thought to be caused by radiotherapy damaging supratentorial structures known to be responsible for cognitive processing. Conversely recent reports demonstrate that non-irradiated patients with tumors of the posterior fossa exhibit similar cognitive impairments to irradiated patients [4,5,8-11].

These studies show that especially children with posterior fossa tumors are at high risk for cognitive or adaptive deficits [5,8,10,12-15].

Accordingly, it seems warranted to summarize the results of investigations on the impact of cerebellar lesions on behavior and cognition in neurosurgical case series and to discuss the findings in the light of recent advances in neuroimaging and its implications for clinicians in form of the hereby presented systematic review.

### Search methods

We selectively searched the PubMed database (http://www.ncbi.nlm.nih.gov/pubmed) for articles containing the terms “cognition” and “cerebellar lesion” with the help of the online search tool from the Endnote X7.2**®** citation program for Mac. All articles published between January 1, 1988 and August 1, 2014 were eligible for further evaluation. All abstracts of the retrieved search results displayed on Endnote X7.2 for Mac were carefully reviewed by the study authors and judged for their feasibility to answer the main research questions: What is the impact of cerebellar lesions on cognition and behavior reported in neurosurgical case series and what are the possible mechanism reported behind this? In case a decision could not be made on the basis of the abstract, the full-text article was downloaded and evaluated. Articles with non-available full-text versions in English language were excluded. Of 125 articles found with initial search, 26 articles were eligible for review inclusion. All further evaluation of included articles was performed on the basis of the full-text version.

The authors state that no explicit review protocol was written and published and no kind of funding or similar support has been received for conduction of this systematic review.

## Review

Most knowledge about the impact of cerebellar lesions on behavior and cognition is derived from reports on pediatric low-grade glioma and medulloblastoma series. Table 1 summarizes behavioral abnormalities and cognition deficits extracted from reports on different neurosurgical case series.

**Table 1:** **Behavioral and cognitive disorders associated with cerebellar lesions reported in neurosurgical case series**

**First Author**	**Year**	**Age of patients in cohort**	**Kind of lesion (Number of subjects)**	**Treatment**	**Observed disorders**
Morgan AT	2011	Pediatric	Pilocytic astrocytoma (n = 7)	Microsurgery, chemotherapy, radiotherapy	Mild dysarthria
			Medulloblastomas (n = 6)		
Aarsen FK	2009	Pediatric	Pilocytic astrocytomas of the cerebellum (n = 29)	Microsurgery, chemotherapy, radiotherapy	Deficits in verbal intelligence, visual-spatial memory, executive functioning, naming and problems with sustained attention and speed.
Puget S	2009	Pediatric	Malignant posterior fossa tumors (n = 61)	Microsurgery, chemotherapy, radiotherapy	Low cognitive performances
De Ribaupierre S	2008	Adult	Various primary and secondary cerebellar tumors (n = 16)	Microsurgery	Severe memory deficits
Kotil K	2008	Pediatric	Medulloblastoma (N = 20)	Microsurgery, chemotherapy, radiotherapy	Cerebellar mutism
			Low grade astrocytoma (n = 12)		
Beebe DW	2005	Pediatric	Cerebellar low grade astrocytomas (N = 103)	Microsurgery	Cognitive and adaptive impairment
Aarsen FK	2004	Pediatric	Pilocytic astrocytomas of the cerebellum (n = 23)	Microsurgery	Apraxia, motor neglect, dysarthria, language, sustained attention, visospatial, executive and memory problems.
					Behavioral disorders: disinhibition, hypospontaneousness, flattened affects, stickiness, anxiousness, rigidity, Asperger disorder, nightmares, posttraumatic stress and attention deficit and hyperactivity disorder.
Gottwald B	2004	Adult	Various primary and secondary tumors (n = 17), Hematomas (n = 4)	Microsurgery	Deficits in executive function, attentional processes working memory and divided attention.
Ronning C	2004	Pediatric	Cerebellar astrocytomas and medulloblastomas	Microsurgery, chemotherapy, radiotherapy	Impaired intelligence, attention, psychomotor speed, verbal memory and visual memory.
Gottwald B	2003	Adult	Tumors (n = 13), Hematomas (n = 3)	Microsurgery	Attention and working memory deficits
Steinlin M	2003	Pediatric	Benign cerebellar tumors (n = 24)	Microsurgery	Significant problems for attention, memory, processing speed and interference. Attention deficits, mutism, addiction problems, anorexia, uncontrolled temper tantrums and phobia.
Silveri MC	1998	Adult	Medulloblastoma of the right cerebellar hemisphere (n = 1)	Microsurgery	Impairment of the immediate retention of verbal information.

Morgan et al. [13] investigated the speech characteristics in a small series of children following resection of cerebellar medulloblastomas and pilocytic astrocytomas. They compared the results of speech assessments in this group to matched healthy controls. They found mild dysarthria in 69% of the patients in the tumor group at long-term follow-up after surgery and concluded that speech deficits may persist even up to 10 years post-surgery.

Ronning et al. [15] studied the neuropsychological profile of young adults treated for cerebellar low-grade astrocytomas and medulloblastomas in childhood. The mean interval from treatment to neuropsychological testing was 14.9 years in the low-grade astrocytoma group and 17.0 years in the medulloblastoma group. They found impaired intelligence, attention, psychomotor speed, verbal memory and visual memory in both groups compared to normal controls, with worse performance in the medulloblastoma group. For that patients in the astrocytoma group were treated with surgery alone, they argued that cerebellar lesions alone could result in long-term cognitive dysfunction.

Kotil K et al. [12] also studied a cohort of 32 children harboring medulloblastomas and pilocytic astrocytomas. They investigated risk factors for the occurrence of postoperative cerebellar mutism. They found cerebellar mutism in 32% of their patients in the early postoperative period. They identified midline localization and vermian incision as significant single independent risk factors for cerebellar mutism and suggested a tumor size of more than 5 centimeters in medulloblastomas as a possible risk factor for cerebellar mutism. Conversely to Morgan et al. [13] who found persisting speech deficits in most of their patients, they argued that cerebellar mutism usually has a self-limiting course and a favorable prognosis, for that they found a return to normal speech in 8 of 10 patients at follow-up examinations.

Aarsen et al. [16] prospectively studied cognitive deficits 3 years after diagnosis in a large series of pediatric patients treated for pilocytic astrocytomas. Children afflicted with pilocytic astrocytomas in all possible supra- and infratentorial locations underwent extensive assessment of intelligence, memory, attention, language, visospatial and executive functions. In a subgroup of patients harboring pilocytic astrocytomas in cerebellar locations they found severe deficits in verbal intelligence, visospatial memory, executive functioning, and naming. Therefore almost 60% of children had problems with an academic achievement. In another study by Aarsen et al. [8] additionally behavioral functioning in children treated for cerebellar pilocytic astrocytoma without additional radio- and chemotherapy was investigated. They assessed speech, language, nonverbal intelligence, attention, memory, executive skills and visospatial functions, as well as neurologic status and neuropsychological functioning in a series of 23 children. The authors found long-term sequelae in all children. Dysarthria, as well as language, sustained attention, visospatial, executive and memory deficits were observed in various combinations and to different degrees. Behavioral disorders were reported in 15 of 23 children; these were: Disinhibition, hypospontaneousness, flattened affects, stickiness, anxiousness, rigidity, Asperger disorder, nightmares, posttraumatic stress and attention deficit and hyperactivity disorder (ADHD). For that the authors found a high percentage of children who needed special education in their study cohort compared to the national average, they concluded that careful long-term neurocognitive follow-up is needed in order to inform parents and teachers about the behavioral and cognitive sequelae and to contribute to timely social and educational intervention.

Data from Steinlin et al. [11] support the findings from Aarsen et al. [8]. They collected long-term follow-up data from a series of children operated for various benign cerebellar tumors without additional radio- or chemotherapy. Although the patients exhibited normal intelligence with a mean IQ of 99.1, a normal performance intelligence quotient of 101.3 and a normal verbal intelligence quotient of 96.8, they found abnormalities in subtesting in 57% of their patients. Extensive neuropsychological testing revealed significant problems in attention, memory, processing speed and interference, as well as visoconstructive problems. Behavioral deficits could be detected in 33% of patients, these were: Mutism, addiction problems, anorexia, uncontrolled temper tantrums and phobia. Involvement of the cerebellar vermis proved to carry an increased risk of neuropsychological and psychiatric problems.

Beebe et al. [5] studied the association of cognitive and adaptive functioning deficits in a large cohort of resected but not irradiated pediatric cerebellar low-grade astrocytomas and examined the effect of tumor location and medical complications on cognitive and adaptive functioning. They proved an elevated risk for cognitive and adaptive impairment in their study cohort, but failed to replicate previous findings of location-specific effects on cognitive or adaptive outcome.

Also Puget et al. [14] found strong cognitive impairment as they correlated the anatomical damage on magnetic resonance imaging and the neurological and neuropsychological deficits in children with malignant posterior fossa tumors. Their study cohort consisted of 61 patients treated with surgery, chemotherapy, and radiotherapy who underwent a detailed neuropsychological evaluation, including a full-scale intelligence quotient several years after the diagnosis. Cerebellar and brain injuries were scored based on the magnetic resonance imaging. They found that neurologic deficits were strong predictors of low cognitive performances irrespective of the other risk factors and the extent of cerebellar deficits and fine motor dexterity impairment were correlated with the degree of damage to the dentate nuclei and inferior vermis. The intelligent quotient scores were also inversely correlated with the severity of the damage to the dentate nuclei. Accordingly it is to be concluded that damage to the dentate nuclei and to the inferior vermis is strongly associated with long-term impairment of neurological and neuropsychological functions.

The study by de Ribaupierre et al. [9] also supports the notion that cerebellar lesions lead to memory deficits independently from the histological entity of cerebellar tumors. They investigated specific effects of various primary and secondary cerebellar tumors on memory in an adult series. Different neuropsychological tests assessing short term and anterograde memory, verbal and visospatial modalities were employed preoperatively. Severe memory deficits in at least one modality were identified in 81% of their patients.

Also Gottwald et al. [4] conducted a detailed neuropsychological examination of 21 adult patients with various primary and secondary cerebellar tumors or hematomas and 21 matched controls. They found deficits in executive functions and in attentional processes such as working memory and divided attention. Furthermore their analysis showed that patients with right-sided lesions were in general more impaired than those with left-sided lesions. They argued that their data supports the hypothesis that lesions of the right cerebellar hemisphere lead to verbal deficits, while those of the left lead to non-verbal deficits [17].

### Cerebellar mutism syndrome

The cerebellar mutism syndrome, also referred to as the posterior fossa syndrome, is a well-described clinical entity that complicates operations for posterior fossa tumors, but may occur in various neurological conditions such as hemorrhage, infection, degenerative disease and neoplastic disease of the cerebellum [12,18]. It is defined as a condition of complete absence of speech that is not associated with other aphasic symptomatology or alteration of consciousness [12]. It typically occurs with a delayed onset of 1–4 days after resection of a cerebellar mass lesion and relatively normal speech in the immediate postoperative course, and subsides in most patients in 1 to 3 months. It is frequently associated with neurologic deficits and neurobehavioral abnormalities. Up to 39% of children operated for posterior fossa tumors develop the syndrome. Although they are alert and cooperative, with normal language comprehension, they are unable to speak and may demonstrate apathy, bladder and bowel incontinence [18].

Permanent sequelae in the form of both motor- and non-motor-related speech deficits are common, especially when the right cerebellar hemisphere is involved [19]. Moderate and severe forms of the cerebellar mutism syndrome are the most frequent types during the initial presentation, and the overall neurocognitive outcome is not as favorable as thought in the earlier publications [20]. In retrospective studies various risk factors have been suggested such as tumor size, length of the vermian incision at surgery, and postoperative complications such as edema within the pontine tegmentum or brachium pontis, hydrocephalus, and meningitis [12].

### Cerebro-cerebellar connections

Disturbances of executive function, impaired spatial cognition, linguistic difficulties, and personality changes, as well as behavioral changes, such as separation anxiety, school phobia, pathologic laughter and aggression in children afflicted with pontine glioma are explained by a disruption of the cerebello-ponto-cerebral circuitry. It is therefore hypothesized that these fibers might modulate behavior and cognition [8,16].

Although cognitive impairment after cerebellar damage has been widely reported, the mechanisms of cerebro-cerebellar interactions are still a matter of debate [21]. The cross-lateralization theory hypothesizes that the cerebellum is strongly interconnected by afferent and efferent fibers with the contralateral cerebral hemispheres. The rate of cerebellar afferents is, compared to the efferents, far higher, which suggests an integrative role for the cerebellum. Higher order cerebral areas, including the dorsolateral prefrontal cortex, as well as the parietal and superior temporal areas, project via the pons to the cerebellum. The feedback loop connects the deep cerebellar nuclei, especially the dentate nuclei, with cerebral areas, via the red nucleus and the thalamus [4].

Impairment of neuropsychological functions from cerebellar lesions is thought to result from disruption of cerebro-cerebellar connections. Disruption of anatomical pathways can also explain the findings in the literature concerning memory deficits in isolated cerebellar lesions [9]. Table 2 summarizes location-specific neuropsychological disorders found in neurosurgical case series. The generally greater impairment of patients with a right-sided lesions are interpreted as a result from the connection of the right cerebellum to the left cerebral hemisphere, which is dominant for language functions and its role for right hand movements [4]. The cross-lateralization theory is supported by most, but not all neurosurgical case series [9].

**Table 2:** **Location of cerebellar lesions and associated neuropsychological disorders reported in neurosurgical case series**

**Location of cerebellar lesion**	**Associated disorders**	**First author**	**Year**
**Vermis**	**Behavioral alterations and executive dysfunction**	**Beebe DW**	**2005**
	**Cerebellar mutism**	**Kotil K**	**2008**
		**Soelva V**	**2013**
	**Increased risk of neuropsychological and psychiatric disorders**	**Steinlin M**	**2003**
**Inferior vermis**	**Low cognitive performances**	**Puget S**	**2009**
**Dentate nucleus**	**Low cognitive performances**	**Puget S**	**2009**
**Cerebellar hemispheres**	**Cerebellar mutism, Speech deficits (more pronounced in right sided lesions)**	**Morgan AT**	**2011**
**Superior cerebellar peduncles**	**Cerebellar mutism**	**Soelva V**	**2013**
		**Van Baarsen KM**	**2013**
		**Ojemann JG**	**2013**

**Figure 1:**
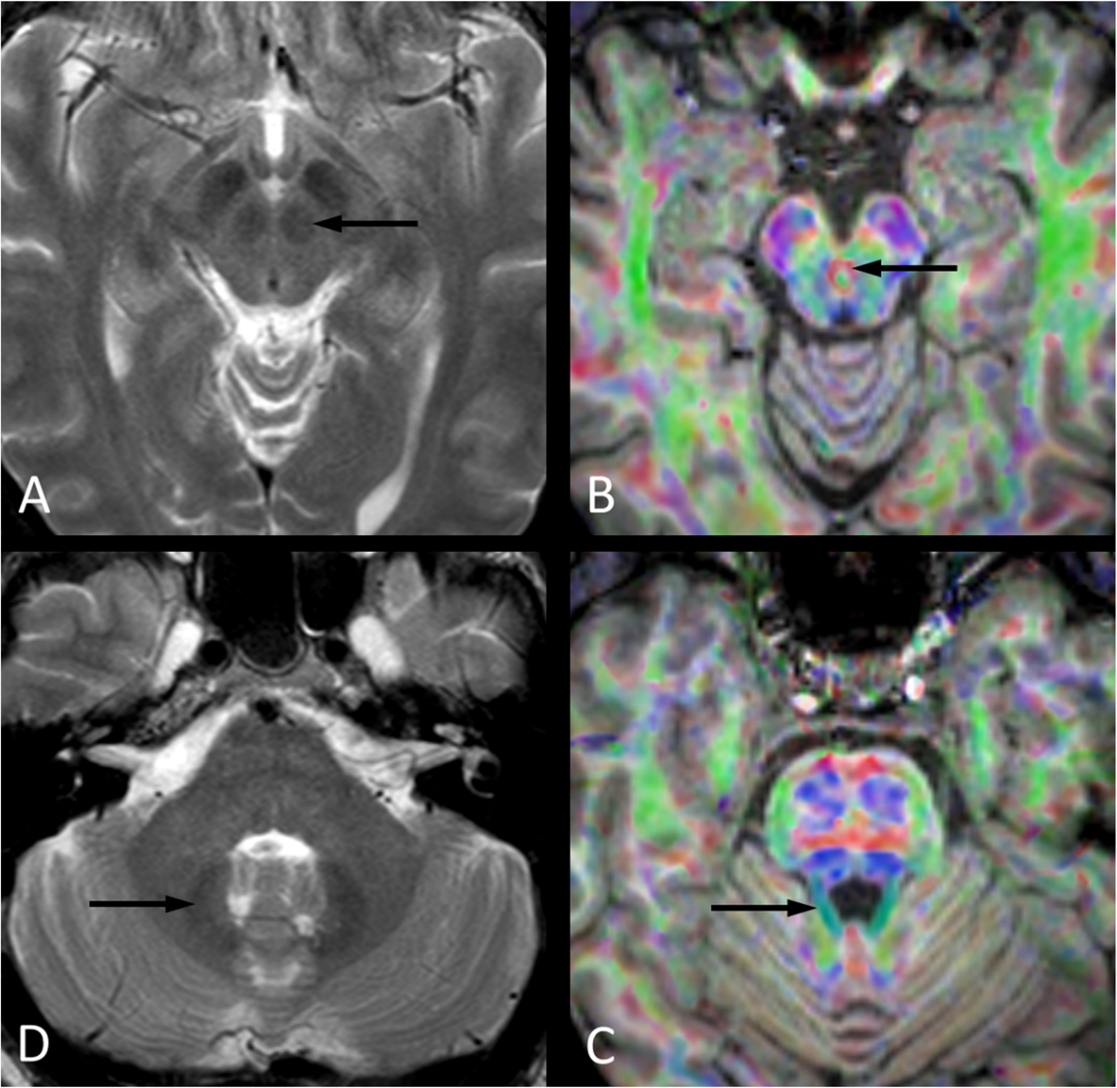
Most important anatomical structures involved in the cerebrocerebellar neurocognitive regulatory system; axial T2-weighted magnetic resonance images at the level of the midbrain **(A)** and brachium pontis **(D)** and cross-sectional color maps derived from diffusion tensor imaging (DTI) with T1-weighted anatomical information at the level of the interpeduncular fossa **(B)** and the pons **(C)**. The cerebellorubral tract connects the dentate nucleus (D, arrow) with the contralateral red nucleus (A, arrow). Its fibers are a component of the brachium conjunctivum (C, arrow) and cross the midline via the decussation (B, arrow) of the superior cerebellar peduncles (brachia conjunctiva) just below the level of the inferior colliculi.

Fronto-cerebellar association fibers are involved in neurocognitive regulatory circuitry, as demonstrated by diffusion tensor imaging (DTI) studies. Figure 1 illustrates the most important anatomical structures involved in the cerebrocerebellar neurocognitive regulatory system using recent MRI techniques.

Soelva et al. [22] studied the fiber tract volumes of fronto-cerebellar association fibers in children following removal of cerebellar tumors with and without cerebellar mutism and in healthy controls. A semiquantitative analysis of fiber tract volumes was employed. It revealed significant diminished values in children exhibiting postoperative cerebellar mutism compared to patients without cerebellar mutism and healthy peers. Differences in fiber volumes were also observed in the superior cerebellar peduncles and midline cerebellar structures in patients with symptoms of cerebellar mutism.

Another DTI study demonstrates that a disruption of the dentate-rubro-thalamic tract, as indicated by an asymmetry of fractional anisotropy in the superior cerebellar peduncles, may cause cerebellar mutism in adults too [18]. In a case report by Gedik et al. [23] a patient exhibiting cerebellar mutism following surgery for a right cerebellar tumor showed right cerebellar and left frontal hypometabolism in 18 F-fluorodeoxyglucose positron emission tomography/computed tomography (FDG PET/CT). Interestingly, the FDG metabolism returned to normal levels as the symptoms resolved.

Ojemann et al. [24] employed DTI to assess the involvement of the dentothalamic tracts within the superior cerebellar peduncles in patients with posterior fossa tumors and the association with posterior fossa syndrome. They found that patients with midline tumors that still had observable superior cerebellar peduncles on DTI did not develop posterior fossa syndrome, whereas in cases of absent superior cerebellar peduncles on DTI patients exhibited posterior fossa syndromes. The authors therefore argued that bilateral injury to the outflow of the dentate nuclei within the superior cerebellar peduncles easily occurs during radical tumor surgery. This is attributed to their highly vulnerable location adjacent to the lateral wall of the fourth ventricle. The importance of the vermis and the dentate nuclei in the cerebro-pontine-cerebellar circuit is also supported by investigations of an animal model conducted by Al-Afif et al. [25]. They split the vermis in juvenile male Sprague Dawley rats and assed locomotor activity, motor coordination, social behavior, and ultrasound vocalization during social interaction. They found that social interaction and vocalization was reduced after surgery in lesioned rats compared to sham-lesioned rats and controls. They concluded that deficient social behavior and vocalization after surgery might be related to vermian splitting in humans as well.

fMRI studies revealed that regions active during overt movement differ from those involved in higher-level functions as language, spatial processing and working memory. Stoodley et al. [1] found that right-handed finger-tapping activated right cerebellar lobules IV, V and VIII, whereas verb generation engaged right cerebellar lobules VI-VIII and mental rotation activated the left cerebellar lobule VII. The authors argued that the cerebellar functional topography identified in this study reflects the involvement of different cerebro-cerebellar circuits depending on the demands of the task being performed. A metaanalysis of functional imaging data conducted by Stoodley et al. [26] shows that sensorimotor processing activates the anterior lobe and parts of lobule VIII. Conversely the activations during cognitive and emotional paradigms are localized to the cerebellar posterior lobe in lobules VI and VII involving both Crus I and Crus II, with no anterior lobe involvement. This supports the theory of an anterior sensorimotor versus posterior cognitive/emotional dichotomy in the human cerebellum.

## Conclusions

The role of the cerebellum in higher cognitive functions beyond coordination and motor control has recently attracted significant interest in the scientific community. The modulatory role of the cerebellum in a fronto-ponto-cerebellar circuit is supported by the fact that neuropsychological disorders are encountered in patients harboring cerebellar lesions independently from the underlying pathology and treatment. In neurosurgical case series especially damage to the dentate nuclei and to the inferior vermis is strongly associated with long-term impairment of neurological and neuropsychological functions. Therefore it is vital to have sound knowledge of the microsurgical anatomy of the cerebellum and dentate nuclei. Advanced neuroimaging techniques contribute to identification of high-risk patients and allow a more effective surgical planning that should focus on maximal tumor resection with minimal risk to important neural structures. Recognition of the cerebellums important role in behavior and cognition is needed to improve the neuropsychological outcome and quality of life for patients afflicted with cerebellar tumors.

Properly designed prospective studies seem warranted to provide stronger evidence regarding effective prevention of the cerebellar mutism syndrome and the best therapeutic approaches with a combination of pharmacological agents and multidisciplinary speech and behavior augmentation.

## Abbreviations

ADHD: Attention deficit and hyperactivity disorder
DTI: Diffusion tensor imaging
FDG PET/CT: 18 F-fluorodeoxyglucose positron emission tomography/computed tomography
fMRI: Functional magnetic resonance imaging
MRI: Magnetic resonance imaging
PET: Positron emission tomography
